# Advanced Gene-Expression Analysis of Skeletal Muscles Focusing on Normal, Glucose-Intolerant, and Diabetic Individuals with Type 2 Diabetes

**DOI:** 10.3390/biomedicines13092181

**Published:** 2025-09-06

**Authors:** Ahmad Barghash, Siba Shanak

**Affiliations:** 1 Department of Computer Science, German Jordanian University, Amman Madaba Street, P.O. Box 35247, Amman 11180, Jordan; ahmad.barghash@gju.edu.jo; 2 Faculty of Sciences, Arab American University, Jenin P.O. Box 240, Palestine

**Keywords:** skeletal muscle, insulin signaling, diabetes mellitus type 2, good health and well-being, differential expression, gene ontology, gene set enrichment, biological pathways, myopathy

## Abstract

**Background:** Glucose intolerance (GI) is a metabolic disorder that is a consequence of hyperglycemia. Glucose intolerance can, under some conditions, progress to type 2 diabetes mellitus (T2D), where insulin is insufficiently utilized. As a result of genetic and lifestyle effects, the incidence of T2D has increased worldwide. Pathophysiological consequences of the disease may include retinopathy, nephropathy, and neuropathy. Skeletal muscle is one of the major organs that regulates blood sugar homeostasis, both at rest and during exercise. Thus, understanding the molecular and genetic perspectives on the contribution of skeletal muscles to the predisposition to diabetes is a hot topic in diabetes research. In this study, we conducted a differential analysis of gene expression and compared the expression profiles of all the genes in the skeletal muscles of normal, glucose-intolerant, and diabetic individuals via the Affymetrix HGU133plus2 platform. Data were collected from the Gene-Expression Omnibus (GEO) series GSE18732. Gene Ontology enrichment and perturbed pathways were thoroughly analyzed. **Results:** We found that genes that were significantly differentially expressed between the different tissues contribute to metabolic pathways related to glucose homeostasis, as well as several signaling pathways related to insulin signaling, e.g., the MAPK, mTOR, Toll-like receptor (TLR), p53, WNT and neurotrophin signaling pathways. Furthermore, some genes related to several malignancies were also differentially expressed across the different clinical groups. Additionally, some of these genes are related to epigenetic regulation. Furthermore, other differentially expressed genes were connected to several myopathies. **Conclusions:** This study may serve as a gene-based analysis that contributes as a basis for further analysis. This investigation may include gene and protein networks that serve in understanding diabetes, the mechanism of action of the involved proteins, and pharmacology and drug design targeting T2D.

## 1. Introduction

Glucose intolerance (GI) is a metabolic ailment that is a consequence of hyperglycemia. Glucose intolerance is frequently referred to as dysglycemia, which involves both prediabetes and diabetes. Moreover, the conditions associated with glucose intolerance include diabetes mellitus (DM), impaired glucose tolerance (IGT), and impaired fasting glucose (IFG) [1].

According to the WHO, in 2019, diabetes was the cause of 1.5 million deaths worldwide [2,3]. Diabetes mellitus (DM) is a chronic disease that is characterized by the progressive failure of pancreatic β-cells. It is now widely recognized that DM progresses in three forms. In the first form, the pancreas is unable to produce sufficient amounts of insulin (type I DM, “here introduced as T1DM”). In the second form, the human system is not capable of using insulin in an effective manner (type II DM “here introduced as T2D”). In a third less common form of diabetes (known as gestational diabetes mellitus (GDM)), diabetes progresses during pregnancy [4].

In healthy adults, blood glucose levels maintain homeostasis at approximately <100 mg/dL (<5.5 mM) with the help of specific hormones. Moreover, skeletal muscles play a major role in blood glucose homeostasis. Studies in healthy individuals have shown that most blood glucose is taken up by skeletal muscles in the postprandial state [5,6]. As a result of postprandial insulin secretion, pathways responsible for restoring blood glucose homeostasis are activated in organs such as the liver, adipose tissue, and muscles. Glucose is transported to skeletal muscle from the extracellular fluid through a membrane transport protein [7]. Two forms of sugar transport proteins are found in mammalian cells: (1) solute carrier family 2 (protein family SLC2), to which glucose transporters (GLUTs 1–14) belong, and (2) solute carrier family 5 (protein family SLC5), to which sodium-dependent glucose cotransporters (SGLTs 1–6) belong [8,9]. Indeed, aerobic exercise and, to a lesser extent, resistance training were found to affect glucose metabolism by increasing hexokinase activity [10], augmenting glycogen synthesis [11,12], and inducing glucose flux through enhancing glycolysis enzymes such as phosphofructokinase [13] and pyruvate dehydrogenase [14]. Recent reviews have also highlighted the broad ecological complexity of the gut microbiome, noting the potential contributions of mycobiome and virome communities in the pathogenesis and metabolism of T2D, via interkingdom interactions [15]. Several studies have emphasized the drug discovery protocols that highlight the importance of strict target identification (e.g., PI3K, PPARγ, AMPK, α-glucosidase, and PTP1B [16,17] in the treatment of diabetes.

Several studies have been carried out recently that link the gene-expression profile in skeletal muscles to the predisposition to diabetes mellitus. Sreekumar et al. focused on the up- and downregulation of many genes in human skeletal muscles. Gene-expression levels were studied before and after insulin treatment [18]. Scott and colleagues [19] reported that protein products of small ANK1 isoforms are linked to T2D through the assembly of the sarcoplasmic reticulum (SR). The SR is involved in glucose transporter 4 (GLUT4) translocation to the plasma membrane and the consequent uptake of glucose. Several genes were found by Barberio and colleagues to be downregulated in T2D patients after gastric bypass [20]. Suh and colleagues investigated the expression profiles of 5000 genes in insulin-sensitive tissues from prediabetic and diabetic Zucker Diabetic Fatty (ZDF) rats at several ages [21]. Voss and colleagues carried out a similar study that shed light on the role of stearoyl-CoA desaturase 1 in insulin resistance [22]. Parikh and colleagues correlated the expression of hundreds of genes with insulin sensitivity in muscle cells [23].

Intensive analysis of perturbed pathways is highly important, especially in diabetes where genes and proteins with high correlations were found to be involved in pathways related to insulin signaling, e.g., fatty acid metabolism and mTOR signaling [17,24]. mTOR, for instance, is a double-edged sword for diabetes [25]. mTOR kinase forms two multi-protein complexes mTORC1 and mTORC [26]. Through serine phosphorylation of insulin receptor substrate 1 (IRS-1) via mTORC1/S6K1 activity, mTORC1 can control glucose homeostasis in different tissues including liver and skeletal muscles [27]. Moreover, instant activation of mTORC1 results in the expansion of β-cell size and mass [28], which affects insulin production. Generally, mTORC1 is labeled as a positive regulator of β-cell functions in response to nutrients [29]. Additionally, the incorporation of rapamycin to inhibit mTORC1 exacerbated hyperglycemia in T2D which supports the mTORC1 roles in the function of pancreas [30]. Moreover, the persistent activation of the mTORC1 signaling pathway has inhibitory effects on the IRS-1, which declines the Akt signaling pathway, leading to noticeable imbalance in the glycolysis pathway in the liver and in the glucose uptake from the blood, which results in glucose intolerance [31]. Similar pathway analysis studies were carried out by Paalsgaard and colleagues [32], who pinpointed the contribution of gene expression in the differential regulation of the insulin pathway. Finally, Moller and colleagues [33] identified repressed markers of autophagy in the skeletal muscle of insulin-resistant patients with T2D.

In this study, we investigated differential gene expression in the skeletal muscles of normal (NGT), glucose-intolerant, and diabetic individuals. To this end, we analyzed the Gene-Expression Omnibus (GEO) series GSE18732, which profiles mRNA expression in the skeletal muscle of normal (NGT), glucose-intolerant (IGT) and type 2 diabetic (DM) subjects [34]. We investigate the effect of up/downregulated differentially expressed genes in this study in a separate manner, as most studies deal with differentially expressed genes in a general manner. We aim to identify the gene network correlated with the progression of glucose intolerance and, consequently, T2D.

## 2. Methods

We chose the NCBI GEO dataset (GSE18732) for this analysis [34]. The dataset profiles gene expression in the skeletal muscles of normal (NGT), glucose-intolerant (IGT) and type 2 diabetic (DM) subjects. Muscle biopsies were obtained from the vastus lateralis, and prepared by Gallagher and colleagues [34] according to the methods described in their study. The groups in this dataset were classified according to the WHO criteria [3], where the DM group was free of antihypoglycemic medications for one week prior to biopsy. Participants visited the laboratory early morning after an overnight fast. The overall design of the Affymetrix dataset included total RNA extracted from the NGT (n = 47, age: 51.3 ± 10.7), IGT (n = 26, age: 56.4 ± 10.7), and DM (n = 45, age: 54.8 ± 10.2) subjects. The fasting glucose level was 5.0 ± 0.4 in NGT, 5.9 ± 0.5 in IGT, and 9.8 ± 4.4 in T2D. The fasting insulin level was 56.6 ± 8.3 in NGT, 88.2 ± 13.5 in IGT, and 91.2 ± 8.9 in T2D. The BMI range was 31.1 ± 7.2 in NGT, 30.9 ± 6.1 in IGT, and 31.4 ± 6.2 in T2D. HbA1c level was 5.5 ± 0.2 in NGT, 5.8 ± 0.3 in IGT, and 7.4 ± 1.8 in T2D. Around two thirds of all samples were from male individuals [34]. We aimed to choose this dataset specifically to study the differential gene expression that can be observed in skeletal muscles as a result of the variance in lifestyle that might lead to glucose intolerance and consequently T2D.

To complete the gene-expression analysis, we performed the methods described in [35,36,37], where we used the KS test in the R-CRAN environment to detect differentially expressed genes in the NGT, IGT, and DM classes. The process involved multiple steps where the initial phase detected the top differentially expressed genes by comparing any two of the three classes. We subsequently checked for the status of the DEGs in which we analyzed the expression changes to report the classes in which the genes were up/downregulated compared with the other classes. The last step was performed to determine the biological significance of the findings, where we defined the perturbed pathways according to the KEGG database (version 2.8.0) [38] in Bioconductor on the basis of irregular gene expression with the help of the human database org.HS.eg. (version 3.21.0) [39]. Moreover, we analyzed and visualized the enriched biological processes (BP) via Gene Ontology (GO) terms via the Bioconductor packages GOSim (version 1.7.1) [40], clusterProfiler (version 4.16.9) [41,42,43,44], and ggplot2 (version 3.5.2) [45].

## 3. Results

In this study, we analyzed the differential expression of genes in skeletal muscle tissue via the GSE18732 series [34], which was hybridized via the Affymetrix HGU133plus2 platform. Analysis was conducted for all genes on the platform. Three main clinical groups were compared in a pairwise manner: normal individuals (NGT), glucose-intolerant individuals (IGT), and diabetic individuals, with diabetes type 2 (DM). Each pairwise comparison was conducted via the KS test, where genes in one of the two groups are downregulated with respect to those in the other group, as well as when the genes in the same former group are upregulated in comparison to those in the other latter groups. The results for the differential expression of all genes between the three groups with a *p* value of 0.01 were filtered and further analyzed. For this list of genes in each group of comparisons, enriched Gene Ontology (GO) terms were calculated. Additionally, perturbed pathways were explored.

### 3.1. Diabetic Group (DM) Compared with the Normal Group (NGT)

First, we compared the diabetic group to the normal group. We began by comparing NGT vs. DM, as it represents the most pronounced contrast in disease state, then examined IGT vs. NGT and IGT vs. DM to characterize the transitional stage. A total of 130 genes were downregulated in the diabetic group, whereas 116 were upregulated compared with those in the normal group (Supplementary Table S1).

#### 3.1.1. Downregulated DM Compared to NGT

**Figure 1:**
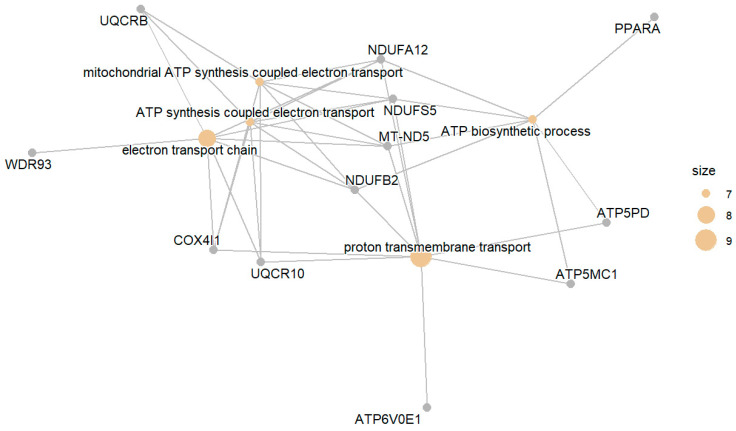
Top enriched GO terms related to gene products downregulated in DM compared with NGT. Circle size corresponds to the number of affiliated genes.

The set of genes whose expression was downregulated in the DM group compared with the NGT group included genes involved in several metabolic processes, most directly linked to sugar metabolism, including the electron transport chain, mitochondrial electron transport, aerobic respiration, and oxidative phosphorylation. Several enriched GO terms were also found for genes that act in biosynthetic processes, e.g., nucleotides and ATP synthesis. Figure 1 presents the top enriched GO terms along with the involved differentially expressed genes.

Perturbed pathways mainly target the degradation of several amino acids and fatty acids, metabolism of pyruvate, glycolysis/gluconeogenesis, signaling of adipocytokine, ketone bodies, glycerophospholipids, the PPAR signaling cascade, calcium signaling, GnRH signaling, the MAPK signaling cascade, the mTOR signaling cascade, the VEGF signaling pathway, TLR signaling, the Fc epsilon RI signaling pathway, and several forms of carcinomas, including pancreatic carcinoma, glioma, and prostate cancer.

#### 3.1.2. Upregulated DM Compared with NGT

**Figure 2:**
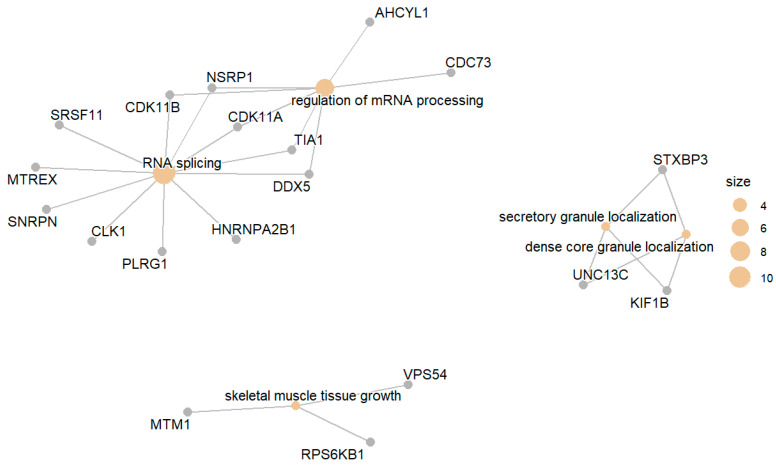
Top enriched GO terms related to upregulated gene products in DM compared with NGT.

The set of genes that were upregulated in the DM group compared with the NGT group included genes involved in processes such as RNA splicing and the regulation of transmembrane transport, e.g., cation transmembrane transport, L-amino acid transport, and secretory granule localization. Figure 2 presents the top enriched GO terms along with the involved differentially expressed genes.

Similarly, perturbed pathways in this set of genes share similar pathways with those in the abovementioned set (where DM is downregulated compared with NGT), such as the metabolism of several amino acids, the MAPK signaling pathway, the mTOR signaling pathway, and the TLR signaling pathway. Moreover, this set includes the Wnt signaling pathway, insulin signaling pathway, TGF-beta signaling pathway and p53 signaling cascade. Additionally, we report pathways related to several cancers, and pathways involved in glycolysis, gluconeogenesis, bile salt and fat secretion, and starch metabolism are enriched. Furthermore, we report perturbed pathways related to development, including oocyte maturation and melanogenesis, in addition to several forms of myopathy, e.g., arrhythmogenic right ventricular cardiomyopathy, dilated cardiomyopathy, and hypertrophic cardiomyopathy.

### 3.2. Glucose-Intolerant Group (IGT) Compared with the Normal Group (NGT)

At this stage, we compared the glucose-intolerant group to the normal group. A total of 107 genes were downregulated in the glucose-intolerant group, whereas only 22 were upregulated compared with those in the normal group (Supplementary Table S2).

#### 3.2.1. Downregulated IGT Compared to NGT

**Figure 3:**
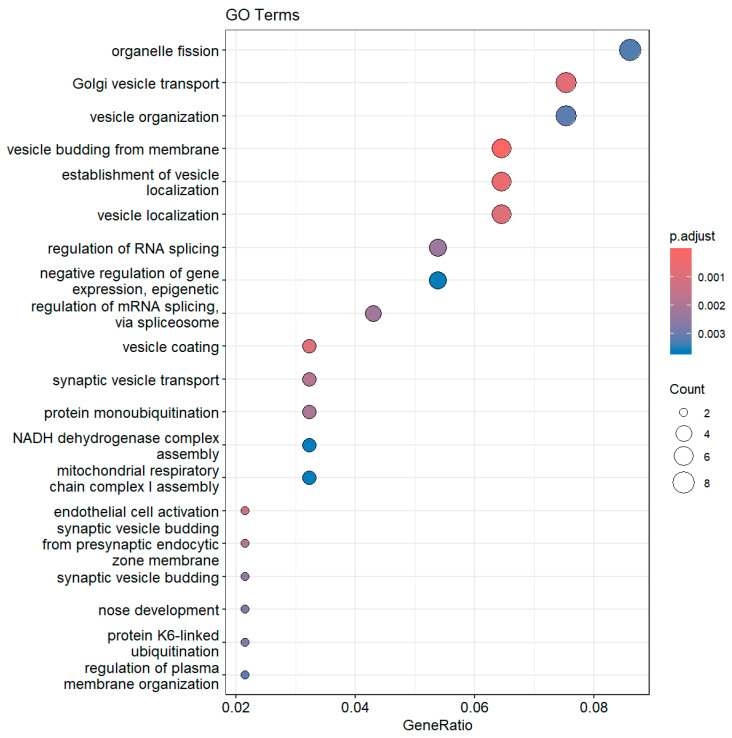
Enriched GO terms related to genes downregulated in IGT compared with NGT. Size corresponds to number of affiliated genes.

**Figure 4:**
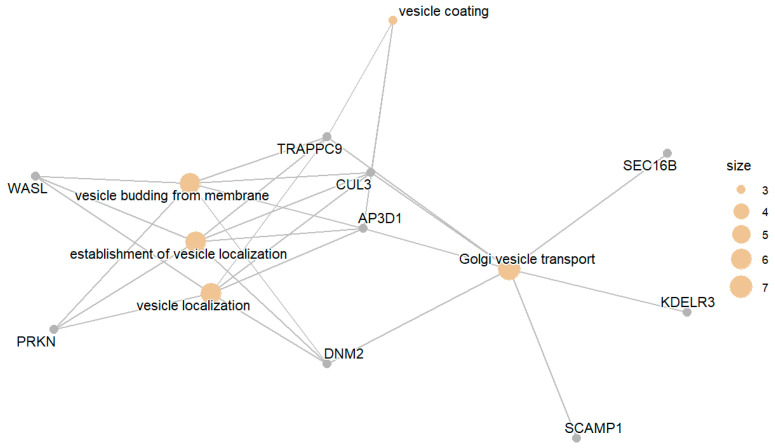
Top enriched GO terms related to gene products downregulated in IGT compared with NGT.

The set of genes whose expression was downregulated in the IGT group compared with the NGT group included genes involved in processes such as Golgi vesicle transport, organization budding from membranes, and localization. mRNA alternative splicing and the epigenetic regulation of splicing were also enriched in the aforementioned group of genes. Figure 3 presents the enriched GO terms, while Figure 4 presents the top enriched GO terms along with the involved differentially expressed genes. Lower adjusted *p*-value denotes a stronger enrichment decision.

The perturbed pathways in this comparison also included the metabolism of several amino acids, sugars, lipids, and drugs by cytochrome enzymes. Moreover, we report signaling cascade pathways, including insulin signaling, PPAR signaling, adipocytokine signaling, calcium signaling, Wnt signaling, VEGF signaling, and chemokine signaling, as well as B-cell and T-cell receptor signaling. Additionally, arrhythmogenic right ventricular cardiomyopathy and dilated cardiomyopathy pathways have also been reported. Because hexokinase II (HK2) is the predominant insulin-regulated hexokinase in adult skeletal muscle, and hexokinase I (HK1) also contributes to total hexokinase capacity [46], we measured both isoforms; notably, HK1 was significantly reduced in IGT when compared to NGT, with pathway analysis indicating matched downregulation of glycolysis. Finally, we report pathways associated with oxidative phosphorylation and T2D.

#### 3.2.2. Upregulated IGT Compared with NGT

**Figure 5:**
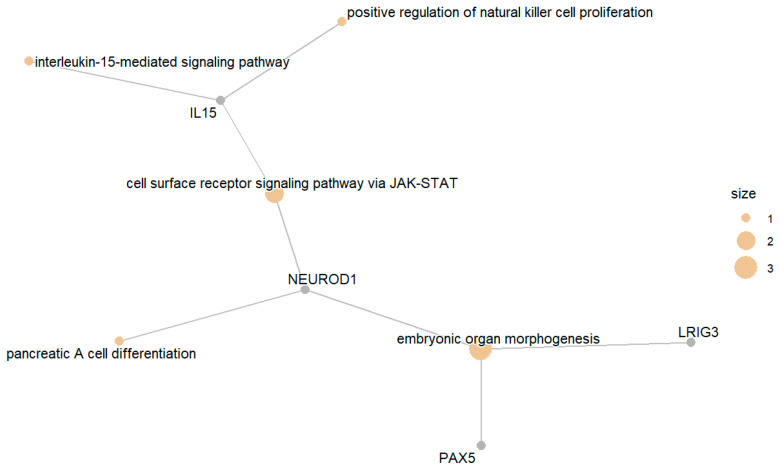
Top enriched GO terms related to upregulated gene products in IGT compared with NGT.

The enriched gene set in this category included a list of genes with GO terms related to diabetes. These genes include those responsible for JAK-STAT signaling, genes involved in type B pancreatic apoptosis, and genes involved in cell-surface receptor signaling via STAT pancreatic A cell differentiation, among other processes. Figure 5 presents the top enriched GO terms along with the involved differentially expressed genes.

Next, we analyzed pathways for this group of genes and found that perturbed pathways are related to neurotrophin signaling, the PPAR signaling pathway, insulin signaling, mTOR signaling, JAK-STAT signaling, T-cell receptor signaling, and Fc epsilon RI signaling.

### 3.3. Diabetic Group (DM) Compared with the Glucose-Intolerant Group (IGT)

Finally, we compared the diabetic group to the glucose-intolerant group. Only 40 genes were downregulated in the diabetic group, whereas 134 were upregulated compared with those in the glucose-intolerant group (Supplementary Table S3).

#### 3.3.1. Downregulated DM Compared with IGT

**Figure 6:**
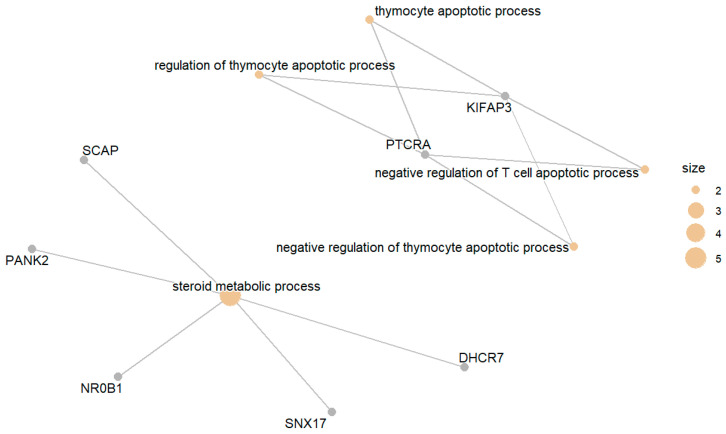
Top enriched GO terms related to genes products downregulated in DM compared with IGT.

The genes in this group were enriched in GO terms related to steroid metabolic processes and the negative regulation of thymocyte apoptotic processes. Figure 6 presents the top enriched GO terms along with the involved differentially expressed genes.

The affected pathways in this group included those related to the metabolism of several amino acids, fatty acids, and glycolipids. Moreover, pathways related to pyruvate metabolism and the citrate cycle are also present in this group. Additionally, we report that signaling pathways include PPAR signaling, adipocytokine signaling, TGF-beta signaling, chemokine signaling, VEGF signaling, TLR signaling, JAK-STAT signaling, T-cell and B-cell signaling, and Wnt signaling.

#### 3.3.2. Upregulated DM Compared with IGT

**Figure 7:**
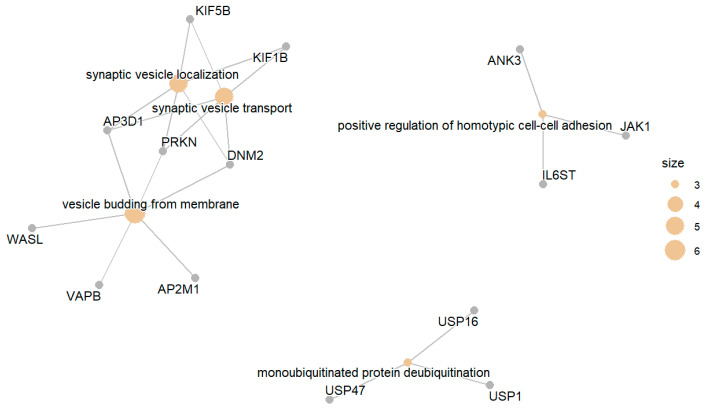
Top enriched GO terms related to upregulated genes in DM compared with IGT.

**Figure 8:**
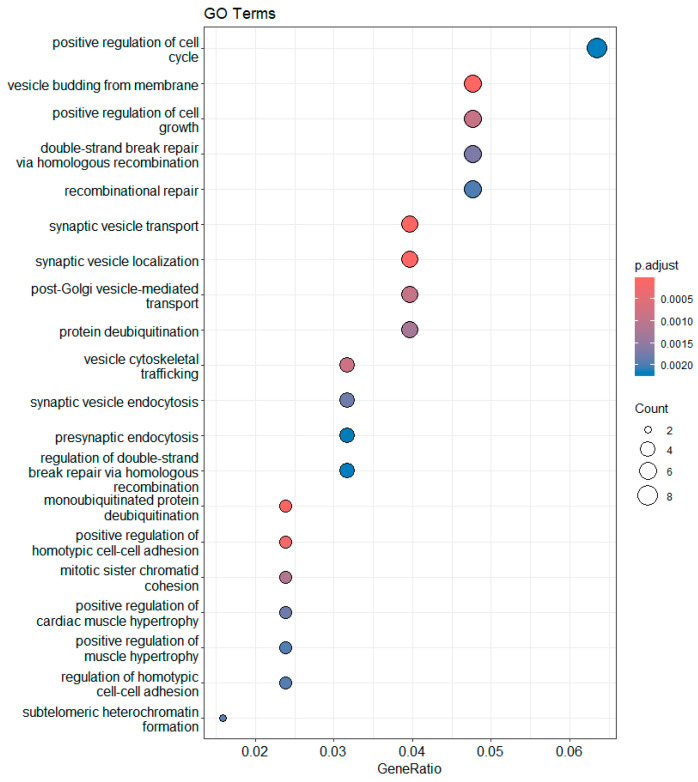
Enriched GO terms related to upregulated gene products in DM compared with those in IGT.

The genes in this group are associated with GO terms related to synaptic vesicle localization, positive regulation of homotypic cell–cell adhesion, and deubiquitination of mono-ubiquitinated proteins. Figure 7 presents the enriched GO terms, while Figure 8 presents the top enriched GO terms along with the involved differentially expressed genes.

Pathways in this group included several signaling cascades, e.g., insulin signaling, adipocytokine signaling, notch signaling, VEGF signaling, and JAK-STAT signaling. Some amino acids and several fat forms (fatty acids and glycolipids) are also metabolized. Table S3 shows the whole set of pathways for this group of genes.

### 3.4. Differential Expression of Protein Targets for the Treatment of Diabetes

Next, we examined the differential expression of a list of genes whose protein products are known as drug targets for the treatment of diabetes (11-beta-HSD1, 17-beta-HSD12, 17bHSD11, GFAT1, PTP-1B, and Sirtuin6) reviewed in [47], and their mode of action is detailed in the indicated paper. None of the listed genes exhibited significant differential expression between any two of the three states.

Furthermore, to validate robustness of the results obtained for the differential expression of genes, we compared datasets analyzed irrespective of sex with those stratified into male and female subgroups. We relied on transcript levels of myosin heavy-chain isoforms (MYH7, MYH2, MYH9, MYH11, and MYH14) as substitute markers of fiber content [48]. This approach provided a practical means to account for plausible variations in fiber-type distribution across samples. Overall, transcriptomic differences remained consistent across both approaches, with the only noticeable deviation observed in MYH2 expression between diabetic females and glucose-intolerant males. These findings indicate that gender-based variability did not significantly change our main conclusions.

## 4. Discussion

In this study, three clinical groups, normal (NGT), glucose-intolerant (IGT), and diabetic (DM) individuals, were compared in a pairwise manner in terms of the differential expression of genes. For the list of differentially expressed genes in each group of comparisons, enriched Gene Ontology (GO) terms and gene pathway analysis were also conducted to understand the genetic basis for the development of diabetes, namely, in terms of molecular function, cellular processes, and pathways.

### 4.1. Diabetic Group (DM) Compared with the Normal Group (NGT)

When the diabetic group (DM) was compared with the normal group (NGT), in which genes were downregulated in DM, metabolic processes linked to sugar metabolism were enriched. These include aerobic respiration processes such as the electron transport chain, aerobic respiration, and oxidative phosphorylation. Additionally, nucleotide and ATP synthesis are enriched. These cellular processes are essential for maintaining blood glucose homeostasis in skeletal muscle, and the lower expression of genes related to these GO terms can directly implicate sugar imbalance in the blood [49]. When the GO terms in this group were compared with the differential expression of genes between the DM and NGT states, where genes were upregulated during DM, they included RNA splicing, cation transmembrane transport, L-amino acid transport, and secretory granule localization. T2D is linked to malfunctioning splicing patterns that affect pancreatic beta cells [50]. L-glutamate transport is associated with overexpressed genes in diabetic tissue. The disrupted stability of these proteins can also contribute to the progression of diabetes [47,51].

Pathways for genes downregulated in DM compared with NGT included several metabolic pathways, e.g., the degradation of several amino acids and fatty acids; the metabolism of pyruvate; glycolysis/gluconeogenesis; and the signaling of adipocytokines, ketone bodies, and glycerophospholipids. The metabolism of amino acids is, on the other hand, enriched in pathways in which genes are overexpressed in diabetic tissues. Similarly, pathways involved in glycolysis, gluconeogenesis, bile salt and fat secretion, and starch metabolism were also enriched in DM tissues.

A comparison of the signaling cascades in both the DM and NGT groups revealed that the MAPK signaling cascade, the mTOR signaling cascade, TLR signaling, p53 signaling, WNT signaling, and neurotrophin signaling pathways are common pathways between the states of genes that are overexpressed in diabetes patients and the genes that are underexpressed in DM patients compared with NGT patients. The MAPK signaling pathway is a secondary branch of insulin signaling [52]. Dysregulation of the mTOR pathway is implicated in insulin resistance [53]. Toll-like receptors (TLRs) are major hallmarks of innate immunity and are thus considered the main contributors to the progression of T2D [54]. Additionally, evidence indicates that macrophage dynamics strongly affect skeletal muscle immunometabolism [55]. During obesity, pro-inflammatory macrophages are recruited into muscle tissue and develop into the M1 stage [56]. These macrophages secrete cytokines that promote JNK and NF-κB pathways. As a result, insulin receptor signaling is interrupted and glucose uptake is reduced [57]. Conversely, M2 macrophages help maintain insulin sensitivity and trigger tissue repair [55]. Thus, a critical link to macrophage-driven signaling between innate immune responses and metabolic homeostasis in skeletal muscle is concluded. The activation of p53 can lead to the progression of insulin resistance and diabetes [58]. The WNT pathway is involved in glucose homeostasis, and mutations in LRP5, the receptor that mediates WNT signaling, may lead to the development of diabetes [59]. Neurotrophin signaling occurs in skeletal muscle, which expresses several neurotrophin receptors. One such neurotrophin is brain-derived neurotrophic factor (BDNF), a major regulator of skeletal muscle metabolism and central metabolic pathways. Low levels of blood BDNF are found in individuals with T2D [60].

Pathways related to several cancers are also present in both groups of genes. Genes related to several forms of carcinoma induce pathway enrichment in several cancer types, including pancreatic carcinoma, glioma, and prostate cancer, among others. Cancer progression and insulin signaling are directly related, where hyperinsulinemia leads to excessive activation of the insulin receptor (IR), which is highly expressed in cancer cells [61].

Pathways in which genes associated with DM are underexpressed compared with NGT tissues include the VEGF signaling pathway. The overexpression of VEGF-A protects against insulin resistance, and insulin stimulates the production of VEGF-A [62]. Vascular endothelial growth factor B (VEGF-B) was recently shown to prevent insulin secretion in MIN6 mice via the phosphatidylinositol 3-kinase-serine/threonine kinase (PI3K-AKT) pathway [63]. Additionally, the Fc epsilon RI signaling pathway was enriched for this group of genes. This pathway is enhanced by insulin signaling in mouse bone marrow-derived mast cells [64]. Compared with NGT, the PPAR signaling cascade is also directly linked to the underexpression of genes in diabetic individuals. The activation of PPAR-gamma in muscle and liver leads to the downstream stimulation of genes involved in insulin signaling and improved insulin sensitivity [65], which explains the involvement of this pathway in differential gene expression. Calcium signaling was also enriched according to the group of genes in this list. Insulin-dependent Ca^2+^ mobilization in skeletal muscle cells and cardiac muscles was recently reported. According to the literature, sarco-endoplasmic reticulum (SER) channels, including the ryanodine receptor (RyR) and the inositol 1,4,5-triphosphate receptor (IP3R), are responsible for the release of Ca^2+^ into the cytosol of skeletal muscles. This action is required for GLUT4 translocation and the consequent uptake of glucose. Additionally, this action stimulates mitochondrial Ca^2+^ uptake in skeletal muscles [66]. The gonadotropin hormone-releasing hormone (GnRH) signaling pathway was also enriched in this group. Recently, autoimmune activation of the GnRH receptor was shown to lead to the development of glucose intolerance and insulin resistance. This consequently causes insulin-stimulated phosphorylation of IRS and a reduction in insulin-stimulated phosphorylation of Akt, which causes a significant reduction in glucose transport via GLUT-4 in skeletal muscles and white adipocytes [67]. Adipocytokine signaling was also enriched in this group. Differential gene expression of the adipocytokine signaling pathway in subcutaneous adipose tissue relative to omental adipose tissue during pregnancy can propagate insulin resistance [68]. T-cell signaling was also enriched in this group of genes. The accumulation of T cells may regulate, via paracrine mechanisms, the metabolic functions of skeletal muscles. This process plays a crucial role in the development of inflammation and insulin resistance [69], and IL-13 controls free glucose production via the inhibition of gluconeogenesis [70].

Pathways in which genes in DM tissues are more highly expressed than those in NGT tissues include the insulin signaling pathway, the TGF-beta signaling pathway, and chemokine signaling. The TGF-β type II receptor was found to be overexpressed in vascular smooth muscle cells induced with high glucose and is thus linked to diabetes [71]. Chemokine signaling has recently been proposed to participate in the progression of diabetic nephropathy [72].

Pathways involved in both the over- and underexpressed states of DM tissues compared with those involved in NGT tissue development include oocyte maturation and meiosis and melanogenesis. Pathogenesis in both states includes hypertrophic cardiomyopathy and dilated cardiomyopathy. Arrhythmogenic right ventricular cardiomyopathy is exclusively overexpressed in DM tissues.

### 4.2. Glucose-Intolerant Group (IGT) Compared with the Normal Group (NGT)

This group of genes included genes involved in processes such as Golgi vesicle transport, organization budding from membranes, and localization. Additionally, alternative mRNA splicing and the epigenetic regulation of splicing were enriched GO terms in the abovementioned group of genes. Golgi vesicle organization is crucial in health, and any corruption in this context is related to disease [73]. Diabetes is also associated with alternative splicing, as well as epigenetics [74]. In addition to transcriptional regulation, epigenetic mechanisms such as DNA methylation have been thoroughly researched in previous studies to shed light on insulin resistance and T2D development in skeletal muscles. It is worth noting that epigenetic alterations can be in part a result of diet and lifestyle. For example, genome-wide methylation studies in muscle and adipose tissue have identified differentially methylated promoters; including PPARGC1A, a vital regulator of mitochondrial synthetic pathway, in T2D individuals [75,76]. Multi-omics analyses were performed in monozygotic twins discordant for T2D. These studies identified matched alterations within skeletal muscle metabolic pathways in gene expression, DNA methylation, and microRNAs [77]. More recently, Do, Shanak, Barghash, and Helms [78] demonstrated that differential exon usage of developmental genes is associated with deregulated epigenetic marks, underscoring the interplay between transcriptional complexity and epigenetic regulation. Together, these findings highlight that our transcriptome-based results are one pivotal part of a more comprehensive regulatory landscape, in which epigenetic modifications play a key role. While this analysis was not assessed in the scope of the present study, integration of epigenomic profiling in future work will be essential to fully unravel the molecular mechanisms driving the transition from normal glucose tolerance to impaired glucose regulation and T2D. On the other hand, the enriched genes in the IGT group were upregulated with respect to those in the NGT group; these genes are responsible for JAK-STAT signaling, type B pancreatic apoptosis, and cell surface receptor signaling via STAT pancreatic A cell differentiation, among other processes. The JAK/STAT signaling pathway is highly conserved and is required for glucose homeostasis. Impaired regulation of this pathway contributes to the progression of diabetes [79]. Additionally, elevated levels of glucose in the serum lead to beta-pancreatic apoptosis in type 2 DM [80].

Pathways in the group of underexpressed genes in the IGT class relative to the NGT state, as in the aforementioned groups, also included the metabolism of several amino acids, sugars, lipids, and drugs by cytochrome enzymes. The signaling cascades included insulin signaling, PPAR signaling, calcium signaling, Wnt signaling, VEGF signaling, chemokine signaling, T-cell receptor signaling, and adipocytokine signaling. All of the mentioned GO terms were also enriched in the group of genes that were differentially expressed between the DM and NGT states. The additional pathways included B-cell receptor signaling. B cells play a role in orchestrating processes related to inflammation and insulin resistance. B cells present antigens to T cells, secrete inflammatory cytokines and produce antibodies. The manipulation of B cells may contribute to preventing the progression of insulin resistance and consequently T2D [7]. Exercise induces the release of several myokines that regulate metabolism and the inflammatory response. An example is the CXCL12 (SDF-1), which has been implicated in muscle remodeling [81]. Furthermore, IL-6 rises sharply during physical activity and functions as an anti-inflammatory mediator [82]. In addition, irisin has been connected to better insulin sensitivity via browning of adipose tissue [83]. Jointly, these exercise-induced factors show the effect of immunometabolic pathways in skeletal muscle in enhancing the metabolic health.

Compared with those in NGT, the genes in IGT are downregulated in IGT compared with NGT insulin signaling, the PPAR signaling pathway, mTOR signaling, and T-cell receptor signaling. The additional pathways included the JAK/STAT signaling, Fc epsilon RI signaling, and neurotrophin signaling pathways, which are associated with the DEGs in the DM and NGT groups.

In addition, pathways that were limited to the underexpressed genes in IGT tissues compared with NGT tissues included several myopathies, i.e., the arrhythmogenic right ventricular cardiomyopathy and dilated cardiomyopathy pathways, as well as the metabolic pathways of oxidative phosphorylation and T2D.

### 4.3. Diabetic Group (DM) Compared with the Glucose-Intolerant Group (IGT)

Compared with those in the IGT group, the genes that were underexpressed in the DM group were enriched in GO terms related to steroid metabolic processes. Despite the current findings that the prognosis of patients with steroid DM is different from that of patients with type 2 DM [84], the current study revealed a shared set of genes that are differentially expressed between diabetic and normal individuals. These genes include SCAP, PANK2, NR0B1, SNX17, and DHCR. Furthermore, negative regulation of thymocyte apoptotic processes was an enriched GO term in this group of genes. The apoptosis of thymocytes has been linked to diabetes and insulin-induced hypoglycemia [84]. Unlike those in the previously mentioned groups, genes that were overexpressed in the IGT group compared with the diabetic group were enriched in GO terms related to synaptic vesicle localization, positive regulation of homotypic cell–cell adhesion, and deubiquitination of mono-ubiquitinated proteins. Diabetes is associated with synaptic vesicle expression and release [85] and with cell—cell adhesion via cadherins and adherins [86]. The deubiquitinase USP36 is upregulated under high-glucose conditions. USP36 deubiquitinates DOCK4 (a mono-ubiquitinated protein), which promotes Wnt/β catenin signaling, diabetic tubular renal injury, and consequently nephropathy [87].

In addition to the aforementioned GO terms, genes in the group that were overexpressed in DM were involved in second messenger signaling cascades and the pentose phosphate pathway. These GO terms are strongly correlated with sugar metabolism. The GO terms for leukocyte and T-cell adhesion and calcium-dependent cell adhesion are related to insulin signaling, as indicated in the abovementioned terms. The reciprocal regulation of cholesterol and low-density lipoprotein biosynthesis are two terms related to central glucose metabolism, e.g., the citric acid cycle. Sterol signaling and insulin are connected via the activation of insulin for sterol-regulatory-element-binding protein 1c (SREBP1c) [88]. Spongiotrophoblast differentiation has been shown to be affected by insulin signaling [89].

The two groups of genes that are differentially expressed between DM and IGT tissues share pathways related to adipocytokine signaling, VEGF signaling, and JAK-STAT signaling, all of which are related to the aforementioned groups of tissue comparisons. The two groups also included pathways related to the metabolism of several amino acids, fatty acids, and glycolipids; pyruvate metabolism; and the citrate cycle. When the genes that were underexpressed in DM compared with those in IGT were compared, the former group included signaling pathways related to the PPAR cascade, the TGF-beta pathway, chemokines, TLR signaling, T-cell and B-cell signaling, and the Wnt pathway. All of these pathways are shared with the previously mentioned pairwise tissue comparisons. On the other hand, pathways exclusive to the latter group included the notch signaling pathway. Notch signaling controls the fate of cells during development. Notch is activated in T2D and promotes diabetic nephropathy [90].

### 4.4. Differential Expression of Protein Targets for the Treatment of Diabetes

Proteins that are drug targets for the treatment of diabetes include 11-beta-HSD1, 17-beta-HSD12, 17bHSD11, GFAT1, PTP-1B, and Sirtuin6; reviewed in [47]. No significant differential expression was found between either two of the three classes. This can ultimately be attributed to the distinct regulatory mechanisms of the proteins involved in maintaining blood glucose homeostasis. The regulation of these proteins seems to occur only at the protein level rather than at the transcriptional level. One another reason can be that the chosen dataset studied gene-expression profiles in skeletal muscles, a rarely tested tissue in T2DM datasets. Moreover, the dataset is uncommon, as it includes multiple subclasses relating to IGT, T2D, healthy individuals, age, BMI, gender, and many others.

The signaling and metabolic pathways identified in this study in skeletal muscle—such as the cascades including MAPK, mTOR, and TLR signaling, as well as glycolysis, oxidative phosphorylation, and fatty acid metabolism—reflect core mechanisms implicated in both adipose tissue and liver perturbations in T2D. For example, the activation of inflammatory mTOR and TLR pathways is likewise fundamental to adipose-driven insulin resistance. On the other hand, the dysregulated mitochondrial function and the consequent unbalanced glucose handling in the liver parallel muscle metabolic malfunctioning [91]. Yet, key distinctions emerge: Adipokine secretion and lipid storage dynamics are notably more conspicuous in adipose tissue than in muscles. Furthermore, hepatic gluconeogenesis is the major player in systemic glucose production—which is not intrinsically mediated by muscle tissues. These tissue-specific roles highlight both common and divergent mechanisms supporting T2D pathology.

Detecting muscle-specific regulatory features introduces several translational possibilities for future research, including clinical decision-making attributes. One possible route is identifying diagnostic biomarkers; these markers help identify subgroups of patients at higher risk of progression to overt diabetes. It additionally helps differentiate stages of the metabolic ailment. Several heterogenous factors might affect the variation in the therapeutic responsiveness of type 2 diabetes, which include exercise, dietary modification, or pharmacological treatment. As a result, personalized interventions may be required.

An important limitation of this study is that the sample composition was determined by the original cohort design, retrieved from a publicly available dataset (GSE18732, [34]). While the diabetic group (n = 45) exhibited a mean HbA1c of 7.4% ± 1.8%, which is consistent with the clinically relevant hyperglycemia, the dataset does not include stratification by disease progression. Consequently, our findings reproduce transcriptomic differential changes in established diabetes but may not completely apprehend changes that result during long-term disease.

## 5. Conclusions

In this study, we identified genes that are differentially expressed in the skeletal muscles of three groups: normal individuals, individuals in the glucose-intolerant group, and individuals in the diabetic group. We presented the effect of differential expression in a different manner where up/downregulated genes are reported separately. In this context, we aimed to understand the genetic basis for the progression of glucose intolerance and consequently diabetes. We found that many of the genes that are differentially expressed among the different clinical groups are involved in central metabolic processes that contribute to blood glucose homeostasis, e.g., glycolysis/gluconeogenesis, fatty acid metabolism, the citric acid cycle, and electron transport chain/oxidative phosphorylation. Interestingly, at all levels of pairwise comparisons of differential expression among the three groups, several signaling cascades are related to insulin signaling or T2D but are not limited to, e.g., the MAPK signaling cascade, the mTOR signaling cascade, TLR signaling, p53 signaling, WNT signaling, and neurotrophin signaling pathways. Several carcinomas have been linked in this study with the progression of T2D, which aligns well with the literature [92]. As formerly stated in the literature, several myopathies were connected in this study to T2D [93]. Indeed, few genes that were differentially expressed in the three clinical groups were found to be linked to epigenetic signals affecting mRNA splicing. This very well matches the findings of previous studies [94]. This study provides a good foundation for further studies in the fields of drug design, target mechanisms of action, pharmacology networks, and whole-genome epigenetic analysis for further understanding of T2D, as well as prevention and treatment of the disease. While our analyses provide a strong bioinformatic discovery framework, future experimental studies, including reporter gene assays, will be essential to validate the functional impact of the muscle-specific enhancers, including the eQTLs identified here.

## Data Availability

The original contributions presented in this study are included in the article/Supplementary Material. Further inquiries can be directed to the corresponding author.

## Supplementary Materials

The following supporting information can be downloaded at: https://www.mdpi.com/article/10.3390/biomedicines13092181/s1.

## Abbreviations

The following abbreviations are used in this manuscript:

T2DM	Type 2 diabetes mellitus
NCBI	National Center for Biotechnology Information
GEO	Gene-Expression Omnibus
DM	Diabetic Group
IGT	Glucose-Intolerant Group
NGT	Normal Group
